# Contemporary Nigerian public health problem: prevention and surveillance are key to combating cholera

**DOI:** 10.3205/dgkh000331

**Published:** 2019-10-31

**Authors:** Israel Oluwasegun Ayenigbara, George Omoniyi Ayenigbara, Rowland Olasunkanmi Adeleke

**Affiliations:** 1 Department of Human Kinetics and Health Education, University of Ibadan, Nigeria; 2 Department of Human Kinetics and Health Education, Adekunle Ajasin University Akungba-Akoko, Ondo State, Nigeria

**Keywords:** cholera, prevention, control, surveillance, public health

## Abstract

Cholera is a public health problem around the world, and it is endemic in Africa, parts of Asia, the Middle East as well as South and Central America.

This review characterizes an cholera outbreak in Nigeria in 2017/2018. On the basis of own experiences and the analysis of historical outbreaks, the *Vibrio* cholera strains, mode of transmission, signs and symptoms, and most important the prevention and control measures are identified. Untreated, the lethality of cholera is up to 70%. Therefore, a multifaceted approach including public policy, surveillance, water purification and hygiene, community sensitization, and the use of oral cholera vaccination (OVC) is vital to prevent, control, and reduce the cholera mortality rate. It is recommended that the government pass legislation to implement preventive and surveillance measures, e.g., invest in drinking water systems, sanitation systems and sewage treatment, and promote public education on basic hygiene. The latter includes boiling and treating water before drinking, washing hands frequently with soap and clean water, thoroughly cooking food before consumption, avoiding open defecation, disposing of wastes properly, and immediately taking anyone with signs and symptoms of cholera such as watery diarrhea to the hospital for treatment.

## Zusammenfassung

Die Cholera ist endemisch in Afrika, Teilen Asiens, dem Mittleren Osten sowie Süd- und Zentralamerika und bedarf weltweit der Berücksichtigung in der öffentlichen Gesundheitssicherung.

Im Review wird ein Choleraausbruch in Nigeria im Zeitraum 2017/2018 charakterisiert. Anhand eigener Erfahrungen und der Analyse historischer Ausbrüche werden die Ätiologie, Übertragung und Krankheitssymptome diskutiert und die Präventions- und Kontrollmaßnahmen identifiziert.

Unbehandelt beträgt die Letalität der Cholera bis 70%. Ihre Prävention erfordert einen vielschichtigen Ansatz, beginnend mit dem politischem Willen, ihrer Surveillance, Trinkwasserhygiene, Sensibilisierung der Bevölkerung und Einsatz der oralen Cholera-Impfung (OVC). Die Regierung muss die Rechtsgrundlage für die Prävention und Ausbruchkontrolle schaffen und Investitionen in die Trinkwasserversorgung und Abwasserbehandlung tätigen. Wasser soll vor dem Genuss gekocht oder behandelt werden einschließlich ordnungsgemäßer Lagerung. Die Hände sollen häufig mit Seife und sauberem Wasser gewaschen werden. Speisen sollen vor dem Verzehr gut gekocht werden. Offene Defäkation soll vermieden werden. Abfälle sollen ordnungsgemäß entsorgt werden. Jeder mit ersten Anzeichen oder Symptomen (wässriger Durchfall) einer Cholera soll sofort hospitalisiert werden.

## Introduction

Cholera is an acute or chronic diarrhea disease caused by the consumption of food or dirty water contaminated with the bacterium *Vibrio cholera* which continues to be a major health risk to the population of many parts of the world. Cholera is currently predominant in developing countries in the tropics and subtropics and is endemic in Africa, parts of Asia, the Middle East as well as South and Central America [1], [2].

As a global health problem, it is a marker of socioeconomic deprivation and absence of social advancement. Epidemiological data revealed that there are 1.3 to 4.0 million cases, and 21,000 to 143,000 deaths annually due to cholera [3]. It causes acute watery diarrhea in children and adults and if left untreated, it can lead to death within hours. Unfortunately, children are the most severely affected [1], [4]. 

Consequently, this review provides detailed information on various ways of preventing the transmission of cholera.

## Cholera in Nigeria

On June 7^th^ 2017, World Health Organization was informed of a cholera outbreak in Kwara State, Nigeria, where it currently remains localized and confined [5]. In addition, between May 1^st^ and June 30^th^ 2017, suspected cholera cases in Kwara State were recorded from five local government areas, namely, Asa (18), Ilorin East (450), Ilorin South (215), Ilorin West (780), and Moro (50) [5]. Furthermore, in June 2017, an aggregate of 1,558 associated cases with cholera have been documented, including 11 deaths (case casualty rate: 0.7%) Thirteen of these cases were confirmed in the laboratory, 50% of the presumed cases are males and 49% are females; the disease affects all age groups [5].

Since the start of 2018, over 5,607 presumed cases of cholera have been reported from nine States [6]. Specifically, Bauchi State has documented 3,757 cases, the largest number in Nigeria to date, followed by Adamawa with 484 cases, Yobe with 449 cases, Borno with 429 cases, Kano with 339 cases, Plateau with 70 cases, and Zamfara with 48 cases. Furthermore, of the 5,607 presumed cases detailed, 98 were confirmed in the laboratory, with 59 deaths. The general case fatality rate (CFR) for all cases was 1.05% and the most affected age groups are 5–14 years (24.8%) and 1–4 years (23.4%), while the male:female ratio is 1.2:1 [6]. Importantly, with the onset of the rainy season, there has been an increase in the number of cholera cases reported in Nigeria, as shown above. This is typical between April and September; the essential reason for this is the contamination of drinking water supplies in villages and rural areas by excreta from infected people [7]. The cholera cases in 2018 in Nigeria are summarized in Table 1 (Tab. 1).

Since the beginning of 2019 until June 30^th^ 2019, a total of 744 suspected cholera cases including 18 deaths (CFR=2.42%) were reported from five states in Nigeria (Adamawa, Bayelsa, Ebonyi, Delta and Kano). Furthermore, during June 2019, 51 samples were collected, 23 tested; 11 were RDT positive and 12 confirmed through stool culture. Also, on the age range of the confirmed cases, 39.7% were aged 1–4 years, while on gender classifications of the suspected cases, 55.6% were females and 44.3% males [8].

## Review on history and current situation of cholera with conclusions to epidemiology, symptoms, prevention and control with focus on Nigeria

### Sources of information

This review focuses on various means of preventing cholera. Literature was recovered from PubMed and Springer Link databases. Furthermore, major international and national health institutions, such as the World Health Organization and Nigeria Center for Disease control, were searched to retrieve relevant information. The year of publication was not a factor as the authors needed to acquire unambiguous data for the topic at discourse. Careful screening was done to ensure that pertinent data were included in this review. Subsequently, all references of the studies included in this review were carefully examined. 

### Brief history of cholera outbreaks

During the 19^th^ century, cholera spread around the world from the Ganges delta in India [1]. Furthermore, six resulting cholera pandemics killed an enormous number of people all over the world [1]. The current (seventh) cholera pandemic began in South Asia in 1961, reached Africa in 1971 and the Americas in 1991 [2].

In Nigeria, reports of cholera outbreaks have not been steady, but the infection is unique because it affects many states. For example, the onset of cholera was obvious in 1970 and in 1991. In the most recent 20 years, three major cholera pandemics have occurred in Nigeria: 1992 [9], 1995–1996 [10], and 1997. The northern states have been known to be endemic for cholera outbreaks in Nigeria. Epidemiological information from the Public Health Department, Kano State Ministry of Health, revealed the recurrence and dispersion of intermittent cholera outbreaks in Kano state from 1995 to 2001, including 2,630 cases in 1995/1996, 847 cases in 1997, and 2,347 cases in 1999 [11]. Furthermore, in Jos, North Central Nigeria, every isolated strain of cholera was *Vibrio cholerae* 01 El tor of Inaba serotype [12].

### Current situation and trends

In 2016, the WHO published worldwide cholera deaths and imported case statistics. 38 countries reported 132.121 cases from all regions, including 17 countries in Africa, 12 in Asia, 4 in Europe, 4 in the Americas, and 1 in Oceania. Haiti, the Democratic Republic of the Congo, Yemen, Somalia, and the United Republic of Tanzania accounted for 80% of all cases. Of cases reported globally, 54% were from Africa, 13% from Asia and 32% from Hispaniola. Imported cases were reported in 9 countries. However, the real number of cholera cases is much higher with an estimated burden of 1.4 to 4.0 million cases, and 21,000 to 143,000 deaths per year worldwide. 

In 2016, reported cases worldwide represented a 23% decrease compared to 2015. This decline, however, is accompanied by more than a doubling of the case fatality rate (1.8% in 2016 vs 0.8% in 2015). Many people, notably in Sub-Saharan Africa and Hispaniola, still die to this disease, thus cholera remains a significant public health problem [13]. 

### Vibrio cholerae strains

Numerous serogroups of *V. cholera* exist, but just two, O1 and O139, have caused outbreaks [2]. For instance, *V. cholerae* O1 has caused every ongoing outbreak in Nigeria. However, although *V. cholerae* O139 was first recognized in Bangladesh in 1992, it had caused cholera outbreaks prior to that. Recently, it has just been distinguished in sporadic cases of cholera outbreaks. Furthermore, it has never been known or discovered outside of Asia [2]. There is no difference in the manifest sickness caused by the two different sero groups of *Vibrio cholerae* [2].

### Mode of transmission

Transmission of cholera is primarily through the fecal-oral route of contaminated food or water caused by poor sanitation [1]. Most cholera cases in developed countries are caused by the consumption of contaminated food while in the developing countries, it is caused by drinking contaminated water [14]. Occasionally, food transmission of *Vibrio cholerae* can occur when individuals collect shellfish. For example, in waters contaminated with sewage, *Vibrio cholerae* accumulates in planktonic crustaceans, which are eaten by oysters and other shellfish [15]. Furthermore, individuals infected with cholera frequently have diarrhea, and infection transmission may occur if the liquid stool, conversationally alluded to as “rice-water”, contaminates water used by others [16]. Also, just one diarrhea stool can cause a one-million increment of *V. cholerae* in the environment [17]. When the diarrhea stool of an infected individual enters public waterways, groundwater or drinking water supplies, contamination and transmission of cholera will occur. As such, drinking any contaminated water, eating any food washed in the contaminated water or eating shellfish living in the contaminated conduit all predispose to becoming infected with cholera. 

### Signs and symptoms

Cholera is a destructive disease that causes extreme and intense water loss. It takes between 12 hours and 5 days for an individual to show symptoms after ingesting contaminated food or water [18]. Furthermore, cholera affects both young and old, and it can kill within hours if untreated. One of the major symptoms of cholera is diarrhea which is often described as “rice water”; it may also have a fishy smell [14]. In addition, an untreated individual with cholera may eliminate 10 to 20 liters of diarrhea daily [14] and serious cholera without treatment could result in life-threatening dehydration and electrolyte imbalances which kill about half of affected individuals. Estimates of the figure of asymptomatic to symptomatic cholera infections range from 3 to 100 [19]. Also, when infected with cholera, a person’s skin may turn bluish-gray from extreme loss of fluids [20], [21]. Fever is not common with cholera, but patients can be fatigued and lethargic, and might have sunken eyes, dry mouth, cold clammy skin, or wrinkled hands and feet [14]. Also, Kussmaul breathing, which is characterized by a deep and labored breathing pattern, can occur as a result of acidosis from stool bicarbonate losses and lactic acidosis associated with poor perfusion [14]. In addition, blood pressure may drop because of dehydration, peripheral pulse is rapid, and urine output diminishes with time. Also, muscle cramping and weakness, altered consciousness, seizures, or coma might occur due to electrolyte imbalances; these are common especially in children [14]. Importantly, many people infected with *V. cholerae* do not develop any symptoms although the bacteria are present in their faeces for 1–10 days after infection and are shed back into the environment, potentially infecting other people [2]. Among people who develop symptoms, the majority has mild or moderate symptoms while a minority develops acute watery diarrhea with severe dehydration [2].

### The WHO cholera road map 2030

In October 2017, thirty-five partners in the Global Task Force on Cholera Control (GTFCC) approved a call to action on ending cholera, an unprecedented engagement to fight cholera through implementation of “Ending Cholera – A Global Roadmap to 2030”. Through the declaration, the GTFCC partners call for a commitment from all stakeholders to support cholera-affected countries and align our energies, efforts, and resources to end cholera transmission [22].

Ending Cholera – A Global Roadmap to 2030 operationalizes the new global strategy for cholera control at the country level and provides a concrete path toward a world in which cholera is no longer a threat to public health. By implementing the strategy between now and 2030, the GTFCC partners will support countries in reducing cholera deaths by 90% [22]. With the commitment of cholera-affected countries, technical partners, and donors, as many as 20 countries could eliminate disease transmission by 2030 [22].

### Implementing the global cholera roadmap

**Figure 1:**
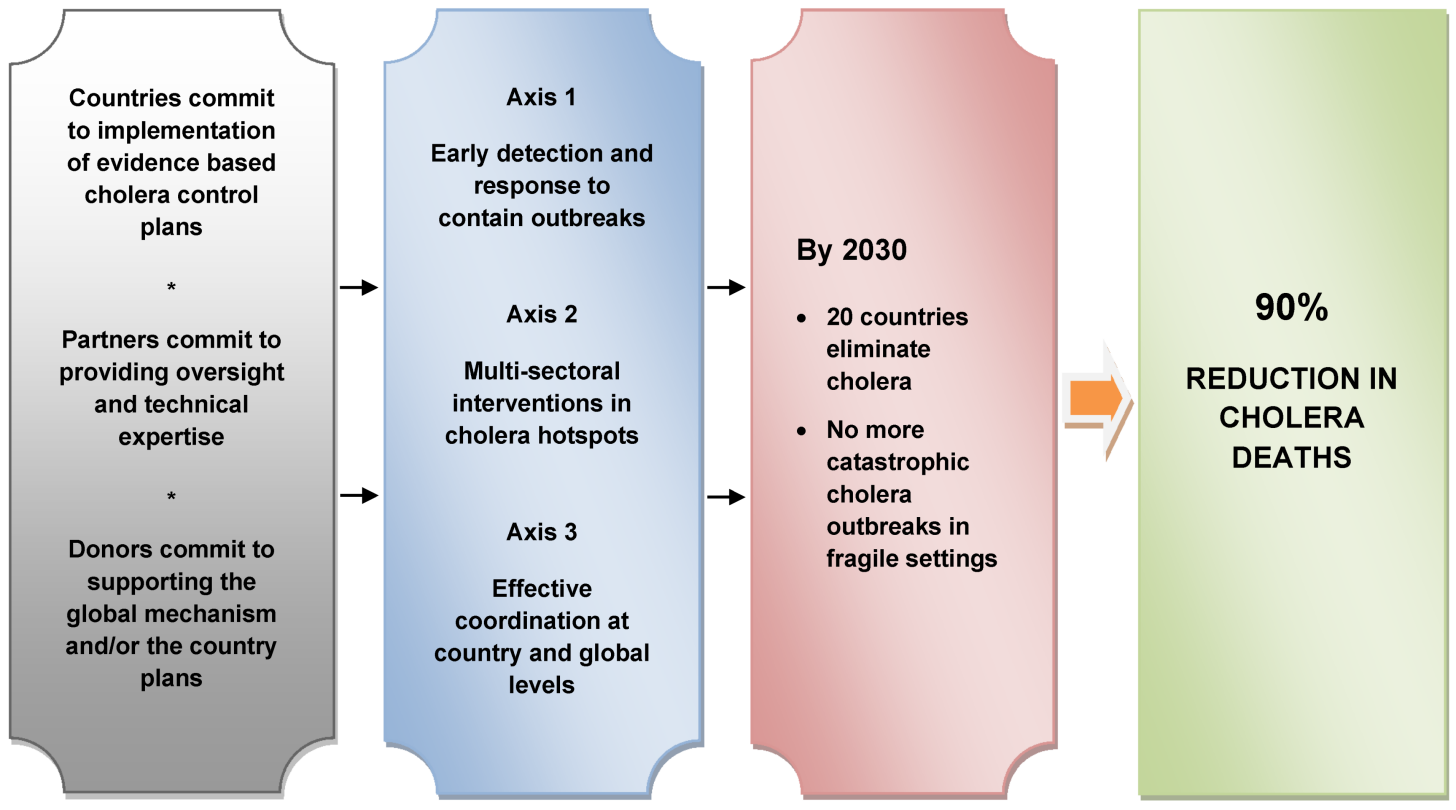
Global cholera roadmap (adapted from [22])

The strategy focuses on the 47 countries affected by cholera today, and consists of multi-sectoral interventions supported by an efficient and effective coordination mechanism. The Global Roadmap focuses on three strategic axes (Figure 1 (Fig. 1), [22]):

1. Early detection and quick response to contain outbreaks: The strategy focuses on containing outbreaks wherever they may occur through early detection and rapid response, which are critical elements for reducing the global burden of cholera. Through interventions such as robust community engagement, strengthening early warning surveillance and laboratory capacities, health systems and supply readiness, and establishing rapid response teams, we can drastically reduce the number of deaths from cholera even in fragile settings.
2. A targeted multi-sectoral approach to prevent cholera recurrence: The strategy also calls on countries and partners to focus on cholera “hotspots”, the relatively small areas most heavily affected by cholera, which experience cases on an ongoing or seasonal basis and play an important role in the spread of cholera to other regions and areas. Cholera transmission can be stopped in these areas through measures including improved hand washing and through use of OCV. In Africa alone, 40 to 80 million people live in cholera hotspots [22].
3. An effective mechanism of coordination for technical support, advocacy, resource mobilization, and partnership at local and global levels: The GTFCC provides a strong framework to support countries in intensifying efforts to control cholera, building upon country-led cross-sectoral cholera control programs, and supporting them through human, technical, and financial resources. As a global network of organizations, the GTFCC is positioned to bring together partners from across all sectors and offers an effective country-driven platform to support advocacy and communications, fundraising, inter-sectoral coordination, and technical assistance.

## Main emphasis on prevention and control

**Figure 2:**
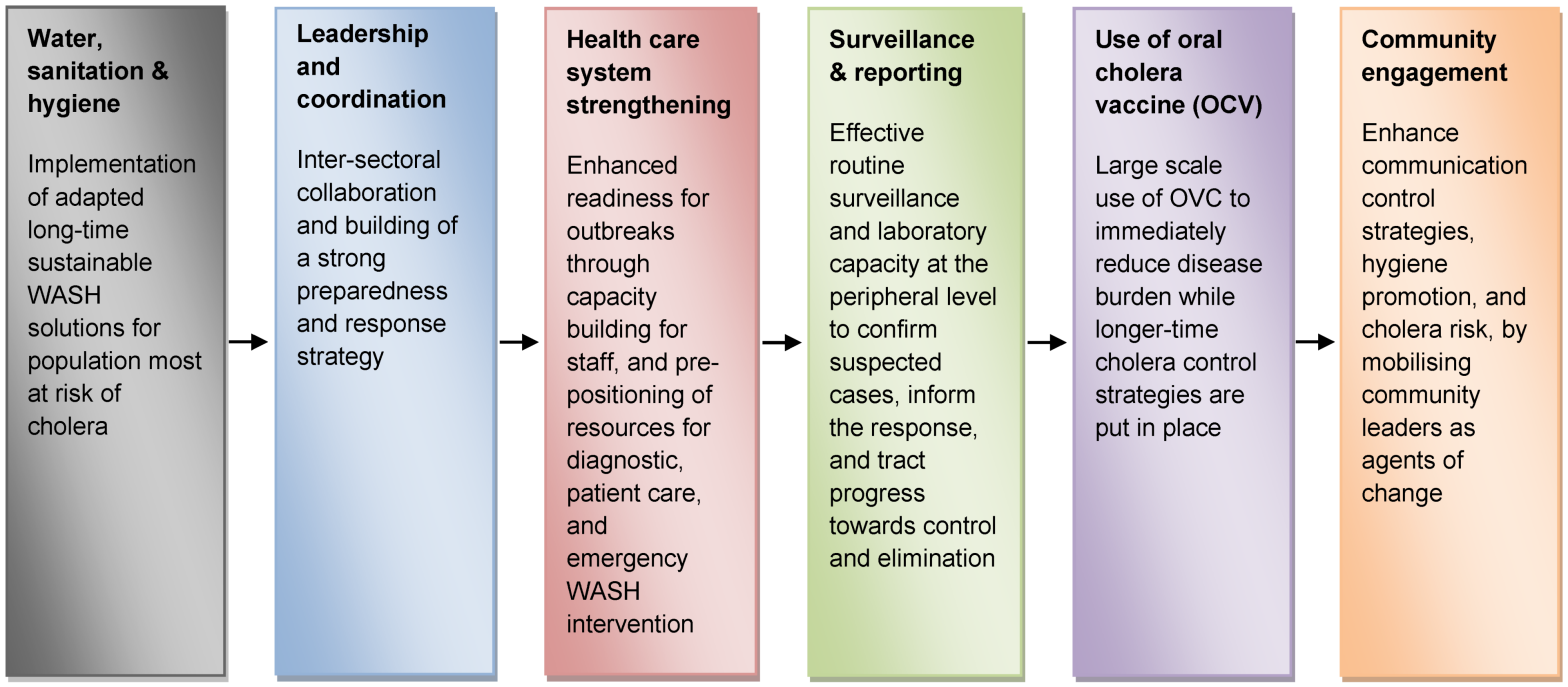
Interventions to prevent and control cholera (adapted from [22])

A multifaceted approach is vital to prevent, control, and to reduce the rate of deaths from cholera. Importantly, a combination of political will on the part of the government, surveillance, water purification and hygiene, social mobilization, and oral cholera vaccination should be used (Figure 2 (Fig. 2)). Although cholera may be perilous and life-threatening, prevention of the disease is normally straightforward if proper sanitation practices are followed [23].

### Political will

First of all, for the successful prevention and control of cholera outbreak, it is necessary to have a political perception and will on the part of the government for risk regulation. Secondly, there is the need for risk regulation and a legislatively based risk approach, e.g., a public health act that each town must have a water treatment facility and a sewage treatment plant.

### Disinfection

Proper disposal and treatment of all materials that may have come into contact with cholera victims’ feces (e.g., clothing, bedding, etc.) are fundamental. These should be cleaned and disinfected by washing in hot water, and using chlorine bleach if possible. Furthermore, hands that touched cholera patients or their clothing, bedding, etc. should be thoroughly cleaned and disinfected with chlorinated water or other effective antimicrobial agents to kill off the bacteria completely.

### Sewage and fecal sludge management

In cholera-affected areas, sewage and fecal sludge need to be treated and managed carefully in order to stop the spread of this disease via human excreta as provision of sanitation and hygiene is an important preventative measure [2]. Open defecation, release of untreated sewage, or dumping of fecal sludge from pit latrines or septic tanks into the environment must be prevented and abolished. Evidently, in many cholera affected regions, there is usually a low degree of sewage treatment [24]. Therefore, the implementation and provision of dry toilets that do not contribute to water pollution as they are not flushed with water may be an important alternative [25].

### Raising public awareness

Massive sensitization and measures to increase public awareness should be carried out in cholera-endemic areas. Warnings about possible cholera contamination should be posted around water sources, with directions on how to decontaminate drinking water (boiling, chlorination etc.) for safer use. Public health education and adherence to appropriate sanitation practices are of primary importance to help prevent and control transmission of cholera and other diseases.

### Water purification

All water used for drinking, washing, or cooking should be disinfected by boiling, chlorination, ozone water treatment, ultraviolet light sterilization (e.g., by solar water disinfection [26]), or antimicrobial filtration in any areas where cholera may be present [23]. Importantly, chlorination and boiling of water are often the least expensive, fastest, and most effective means of halting cholera transmission. Cloth filters or sari filtration, though very basic, have significantly reduced the occurrence of cholera when used in poor villages in Bangladesh that rely on untreated surface water [23]. Better antimicrobial filters, like those present in advanced individual water-treatment hiking kits, are most effective. 

### Hand washing

Proper hand washing with soap and clean water after using a toilet and before handling food or eating is also recommended for cholera prevention as this will help kill cholera bacteria and other pathogens on the hands [23], [27].

### Surveillance

Surveillance and prompt reporting is very important in the control and prevention of cholera because it allows rapid containment of cholera epidemics [2]. Cholera exists as a seasonal disease in many endemic countries, occurring annually and mostly during rainy seasons. As such, proper surveillance systems provide early alerts to outbreaks, enabling coordinated and fast-track response as well as development of preparedness plans. Furthermore, efficient surveillance systems can also improve the risk assessment for potential cholera outbreaks. Also, understanding the seasonality and location of outbreaks provides guidance for improving cholera control activities for the most vulnerable [2]. For prevention to be effective, it is paramount that cholera cases should be adequately reported to local and national health authorities.

### Vaccination

A number of safe effective oral vaccines for cholera is available [1], [28]. The World Health Organization has three prequalified oral cholera vaccines (OCVs), namely Dukoral, Sanchol, and Euvichol. Dukoral, an orally administered, inactivated whole cell vaccine, has an overall efficacy of about 52% during the first year after administration and 62% in the second year, with minimal side effects [1], [28]. It is available in over 60 countries around the world. Furthermore, more than 15 million doses of OCVs have been used in mass vaccination campaigns. The campaigns have been carried out in areas experiencing an outbreak, areas of heightened vulnerability during humanitarian crises, and among populations living in highly endemic areas known as “hotspots” [1], [2].

### Recommendations

Based on this review, the following recommendations are made:

1. Political will on the part of the government is needed to create a legal basis for fighting cholera, for instance, by investing in drinking water systems, sanitation system and sewage treatment plants. This could be achieved through enacting laws that would improve and provide clean water for all.
2. Ensure water is well boiled and treated before drinking; also, bottled water must be properly sealed. Furthermore, boiled and treated water should be stored in a clean and safe container.
3. Personal hygiene should be constant and paramount. Hands must be washed frequently with soap and clean water or alcohol-based hand sanitizer should be used if soap and water are not available.
4. Ensure all food is well cooked before consumption. Avoid eating fruits and vegetables in raw form, except after washing them in clean water or peeling them yourself.
5. Avoid open defecation and indiscriminate refuse dumping and ensure proper disposal of waste and clearing of sewage.
6. Upon any signs and symptoms of cholera such as sudden watery diarrhea, a health care facility should be visited immediately.

## Conclusion

Cholera is a deadly disease. A multifaceted approach of political will on the part of the government, surveillance, water purification and hygiene, community sensitization, and oral cholera vaccination are vital to prevent, control, and to reduce the rate of deaths from cholera. Furthermore, public health education and adherence to appropriate and basic sanitation practices, for example hand washing, are of primary importance to help prevent and control transmission of cholera and other diseases.

## Notes

### Acknowledgments

Great thanks are given to unknown reviewers for valuable comments.

### Competing interests

The authors declare that they have no competing interests.

## Figures and Tables

**Table 1:**
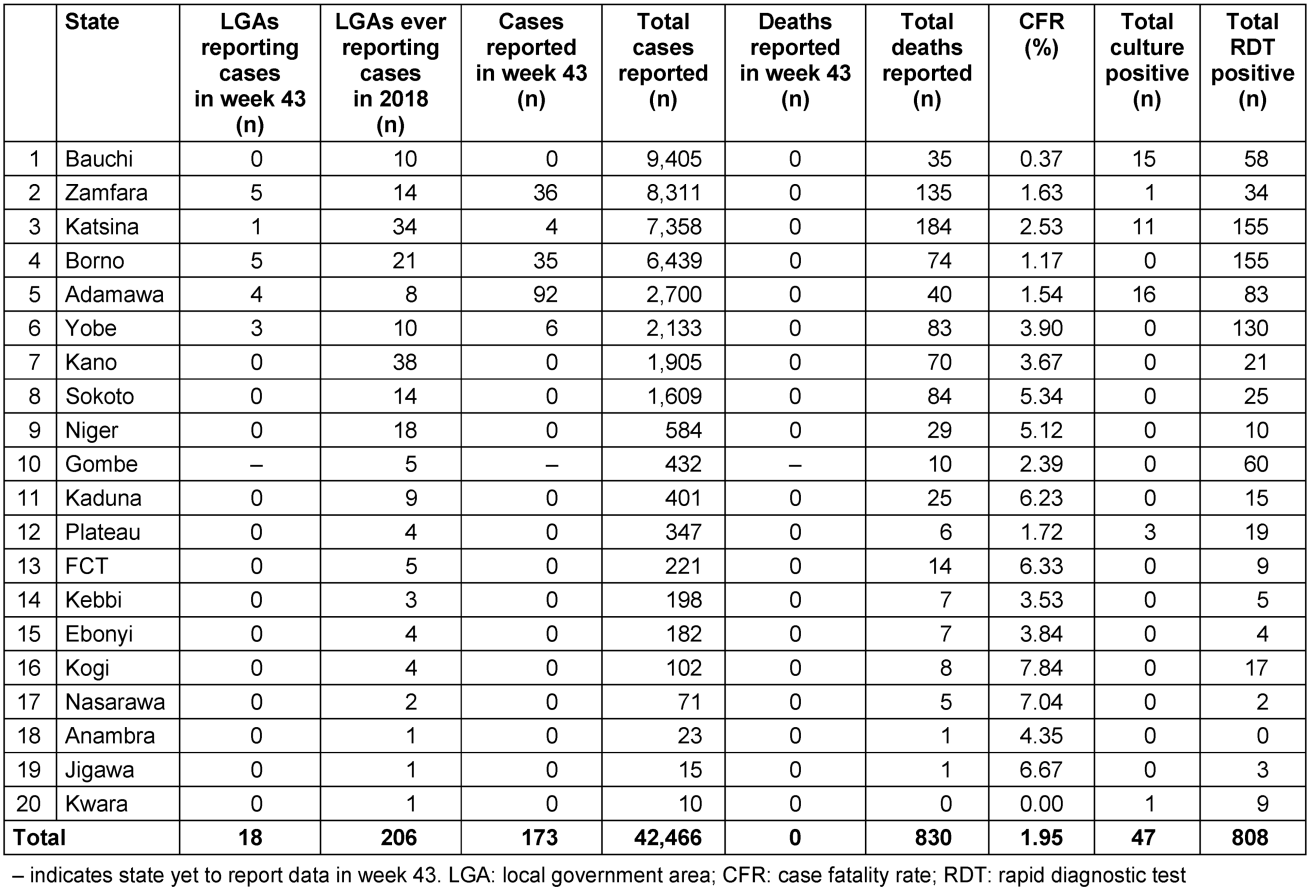
Reported suspected cholera cases by states in Nigeria, epidemiological week (43) end of 2018 [Data source: Nigeria Centre for Disease Control]
